# Access to Technology-Mediated Community Mental Health Care Among Low-Socioeconomic Status Consumers With Serious Mental Illness: Qualitative Study

**DOI:** 10.2196/79608

**Published:** 2026-04-20

**Authors:** Alicia K Stone, Tiffany C Veinot

**Affiliations:** 1 School of Information, University of Michigan, 105 S. State St, Ann Arbor, MI, 48109, United States; 2 Department of Health Behavior and Health Education, School of Public Health, University of Michigan, Ann Arbor, MI, United States; 3 Department of Learning Health Sciences, School of Medicine, University of Michigan, Ann Arbor, MI, United States

**Keywords:** health care equity, access to care, telehealth, digital health, mental health

## Abstract

**Background:**

Access to mental health care is critical for the effective management of serious mental illness (SMI), but consumers with low socioeconomic status (SES) have lower rates of service usage and worse retention in care. Digital technologies are often lauded as a way to bridge access gaps; however, little is known about how technology-mediated care may influence care access among low-SES consumers and how consumers use technology in care access.

**Objective:**

This study aimed to examine the applicability of Levesque et al’s access framework to technology-mediated care for SMI and analyze how low-SES consumers use technology to facilitate care access. Furthermore, the study assesses whether and how technologies are involved in care access at multiple points within the process of accessing care.

**Methods:**

This study used 2 qualitative methods: ethnographic observations at a mental health treatment court and interviews with low-SES consumers with SMI using community mental health care (n=14) and key informant interviews with health and service providers working with this population (n=14). Observations occurred from July 2022 through September 2023, and interviews occurred between January 2022 and May 2024. Data analysis involved both inductive and deductive coding approaches. Data from both the interviews and observations were analyzed in NVivo and further triangulated through analytic memos.

**Results:**

Levesque et al’s framework required several extensions to accommodate technology-mediated care related to SMI for low-SES consumers: (1) a cyclical rather than linear trajectory; (2) simultaneous care acquisition from multiple health and service providers; (3) staying in care long-term; (4) identification of both one-time and ongoing health needs; and (5) an emergency pathway for entering care. Consumers often faced challenges related to the varied digital requirements of each provider and a dearth of integrative, patient-facing tools like portals. Within this context, some consumers use mobile apps, communication, and telehealth technologies across various care access stages. Consumers used technology by figuring out how to navigate technology-mediated care, especially by leaning on others, such as case managers, for support. These others provided consumers with temporary technologies, showed them how to use technologies, and accompanied them through the process of using technology for accessing care.

**Conclusions:**

This study highlights that accessing care is iterative and ongoing, involving multiple forms of co-occurring service provision. A theoretical contribution of this work is its extension of Levesque et al’s care access framework to better reflect technology-mediated care for SMI among low-SES consumers. This work also underscores ongoing challenges for accessing technology-mediated care and the importance of human support in addressing access difficulties. Clinical implications include incorporating digital readiness assessments and providing comprehensive guidance on how consumers can effectively use technologies for care. Future work should investigate how technology-mediated care can make care access easier rather than harder.

## Introduction

Serious mental illness (SMI)—a set of chronic conditions affecting 1 in 25 adults in the United States—significantly impacts individuals’ abilities to function in their daily lives [1]. SMI care is truly a matter of life and death, as people with SMI are at an increased risk of suicide [2]. Moreover, people with SMI who are using mental health care have better health outcomes overall [3].

Nevertheless, the rate of mental health care use in people with SMI in the United States is insufficient to adequately manage their mental health [4]. Indeed, fewer than two-thirds of adults with SMI (henceforth, “consumers”) in the United States received mental health treatment in the past year [5]. Moreover, a lack of retention in mental health care is common: up to one-third of those who have some contact with mental health services disengage from care [6]. For our purposes, nonretention in care is defined as disengaging from treatment without symptom improvement or not completing treatment for the recommended duration [7].

Care usage and retention among consumers are also differential; those with low socioeconomic status (SES)—“the socially derived economic factors that influence what positions individuals or groups hold within the multiple-stratified structure of a society” [8]—have the lowest mental health care usage rates [9]. Furthermore, consumers with low SES have higher rates of care dropout [10]. Barriers to care are also amplified and compounded among consumers with low SES, which impedes care retention [11]. Such barriers include the stress associated with multiple competing obligations, like changing work schedules, transportation precarity, and childcare needs [11]. Consumers who drop out of care are at greater risk of experiencing homelessness, hospitalization, and negative health outcomes such as suicide [3,12].

Furthermore, consumers with low SES who are not in care may have increased interactions with the criminal justice system, resulting in disproportionately high rates of imprisonment [13,14]. Racial discrimination that results in the overrepresentation of African American and Indigenous people in US prisons exacerbates the issue for low-SES consumers with SMI in these minoritized racial and ethnic groups [14,15].

Usage of mental health care first requires access to that care, which we define as “the opportunity to identify healthcare needs, to seek healthcare services, to reach, to obtain or use healthcare services, and to actually have the need for services fulfilled” [16]. Access is at least partly a result of how mental health care systems function, or their “arrangements.” Notably, mental health care arrangements for consumers who have low SES often differ from those of their higher SES counterparts. Specifically, care for low-SES consumers is predominantly offered through a public, Medicaid-funded system [17], known as “community mental health care,” rather than being funded out-of-pocket or through private health insurance. The American Psychological Association defines community mental health care (CMH) as “providing prevention, treatment, and rehabilitation mental health services, sometimes organized as a practical alternative to the largely custodial care given in mental hospitals” [18].

CMH services are offered through local entities that provide counseling, psychiatric care, and case management, while also facilitating access to critical social services for those with low-SES, such as affordable housing and food purchasing assistance. CMH services can successfully support low-SES consumers with SMI, resulting in greater acceptance of health care, fewer hospital admissions, cost reductions compared with hospital-based outpatient care, and fewer deaths by suicide [19]. CMH also provides specific services to consumers with criminal justice involvement, including re-entry programs for people who are on probation or leaving incarceration. Moreover, and critically for this research, CMH provides health care via specialized courts that divert people with SMI and criminal justice involvement away from prison. Such courts typically require that people with SMI use mental health care as a part of probation conditions, a structured mode of enhancing care access for consumers that provides a rich opportunity for investigating how access to care is accomplished in an increasingly technology-mediated care environment.

Of note, technology is often lauded as a way to enhance access to mental health care [20]. This study defines technology broadly as the “tools, techniques, crafts, systems, or methods of organization, to solve a problem or serve some purpose or end” [21]. While digital technologies are vital to modern care access, the expanded definition allows us to analyze different forms of technology (like pen and paper, physical calendars, calendaring apps, patient portals, etc) to understand their role in care access [21]. Digital health technologies can increase care access by facilitating remote care [22], enabling remote monitoring [23], and improving the referral process [24] while decreasing costs for low-SES consumers. Nevertheless, little is known about how care access processes may change in such an environment, and for low-SES consumers.

In theory, technologies could also facilitate retention in mental health care. Reminders like text messages and automated phone calls, for example, can help people remember scheduled appointments [25]. The use of communication technologies to provide health care remotely, called “telehealth” [26], also improves treatment retention in some, but not all, cases [27-30]. Retention outcomes for telehealth may be enhanced when providers establish care via in-person visits and then transition to telehealth [31]. However, few studies specifically examine care retention in CMH and criminal justice contexts. Moreover, there is a dearth of research characterizing how consumers with low SES use technology to enable access to and retention in mental health care. These issues are critical because low-SES consumers have lower device ownership and face more difficulties with using technologies [32-34].

Thus, this study seeks to understand the theoretical implications of technology-mediated CMH care to better understand its role in the process of accessing care.

## Methods

### Overview

The study used two qualitative methods: (1) ethnographic observations and (2) semistructured interviews. Observations occurred at a Mental Health Treatment Court (“the court” or MHTC). The court links consumers with SMI who have experienced recent legal trouble—most of whom have low-SES—to health care specifically for their SMI instead of prison. Interviews were conducted with both low-SES consumers (C) with SMI (n=14) who had some form of prior criminal justice contact and health care and service providers (HSPs) working with this population (n=14) who served as key informants regarding study research questions. The methodologies were reported in accordance with the COREQ (Consolidated Criteria for Reporting Qualitative Research) checklist.

### Ethical Considerations

The Institutional Review Board at the University of Michigan reviewed this study and determined that the studies (observations and service provider interview study HUM00205027; consumer interview study HUM00238889) were exempt from ongoing review based on minimal-risk research with adults. To protect confidentiality, participants were assigned a participant code. After reviewing the study information, all consumers and HSP participants provided verbal informed consent to participate in interviews. All HSP participants were offered a US $25 incentive for a 60-minute interview, and consumers a US $40 incentive for a 90-minute interview. This study was executed in accordance with the principles of the Declaration of Helsinki.

### Conceptual Model: Access to Health Care

The present research is framed using the influential “Conceptual framework of access to health care” of Levesque et al [16] to conceptually outline the process of accessing mental health care. The general framework of Levesque et al [16], intended to apply across varied clinical contexts, establishes five stages of health care access: (1) perception or identification of health care needs, which is followed by (2) health care seeking, (3) health care reaching, (4) health care usage (primary and secondary access, which vary in type and intensity), and (5) health care consequences (economic, satisfaction, and health). At the same time, organizational and individual factors that enable access precede each access point. In the model, individual consumer characteristics also influence the process of health care access. Hence, this framework centers the process of accessing mental health care, which is well-suited to achieving study objectives. However, to extend the model to reflect the chronicity of SMI, the study includes retention in care as part of the guiding framework.

Beyond this, it is notable that the Levesque et al [16] model is for general health care, not mental health care, nor does it include necessary technological factors. It also does not consider how access to care may differ for those with low-SES. Therefore, by addressing the study’s research questions, this study aims to enhance and extend the Levesque et al [16] model to suit empirical data as needed.

Using the Levesque et al [16] framework, with the addition of retention in care as an access stage, this study addresses the following research questions:

- RQ1. What changes, if any, are necessary to improve the applicability of the conceptual framework of access to health care related to SMI for low-SES consumers and technology-mediated care contexts?
- RQ2. How do low-SES consumers use technologies throughout the process of accessing health care related to their SMI?

### Research Setting

This research was conducted in one Midwestern state from January 2022 through May 2024, involving ethnographic observations at one court in a Midwestern state. The court is a collaboration of multiple care and service providers that requires that consumers with SMI who participate, most of whom lack employment and have public insurance, stay in health care for their SMI for at least 12 months. Such requirements must be met, or a person with SMI could face incarceration or altered judicial supervision for the offenses that first brought them into contact with the criminal justice system.

Consumers at the MHTC primarily receive mental health care through a local CMH agency with additional support from local nonprofit agencies, but the court monitors their overall progress. MHTC review sessions occur at the court multiple times monthly with multiple staff HSPs from CMH, nonprofit organizations, and criminal justice sectors. Many HSPs interact with consumers remotely between review sessions via email, phone calls, and text messages. HSPs also contribute to regular, in-person court team meetings to monitor and assess participants’ progress with health care for their SMI and related social services.

During the early COVID-19 pandemic, MHTC conducted review sessions remotely via videoconference, with in-person meetings resuming in late 2022. Since consumers enrolled in the court typically face multiple challenges in accessing and staying in mental health care, studying this setting offers a valuable opportunity for identifying how technology access may help or hinder access to SMI-related health care and related services among low-SES consumers.

### Recruitment and Sampling

While the court review sessions were open to the public, the first author (AKS) secured support from stakeholders before the observational period. The first author was a volunteer with a mental health nonprofit that supported the MHTC participants, so they started discussing the proposed research with the nonprofit board of directors to get their feedback and ensure no conflicts of interest. After multiple discussions, the board of directors communicated its project support. Then, the first author obtained permission from the MHTC judge to observe the court. To do so, the first author presented the judge with the research protocol and Institutional Review Board information and held a web-based meeting to discuss the research aims before the onset of observations. Observations were conducted for the full length of most court review sessions from July 2022 through Sept 2023. AKS recruited HSPs linked to the MHTC via a purposive sampling approach to represent different consumer-facing roles [35]. Participants and stakeholders were made aware of AKS’s presence as a researcher via an announcement from the judge, in which they described the research goals, interest in conducting said research, and rationale for the observations.

The first author recruited consumers in person at one MHTC (all receiving care through CMH) and consumers receiving CMH care with criminal justice histories through local mental health clubhouses. Starting with a referral-based strategy, a member of a mental health nonprofit provided the initial connection to a clubhouse member, who subsequently connected the first author with the staff leadership of that organization. After the first set of consumers and peer support specialists were interviewed from the clubhouses, the study used chain referral sampling to interview other members across the state.

Clubhouses are intentionally formed as nonclinical spaces for individuals with SMI to build community and support recovery [36]. Clubhouses adopt a psychosocial rehabilitation model prioritizing teamwork and a strengths-based focus for “members.” Many clubhouses throughout the state, including the ones from which participants were recruited, require members to be consumers at their local CMH. Notably, some consumers play a dual role as formal peer support specialists. Services such as CMH typically use peer support specialists, and this group often bridges other mental health services, like these mental health clubhouses.

### Data Collection

#### Observations of Court Review Sessions

The first author conducted ethnographic observations during biweekly court review sessions from July 2022 to September 2023, including the transition from video conferences due to the COVID-19 pandemic to in-person sessions. The observations averaged over 1 hour and 40 minutes per session. The lead author observed 23 of the 28 sessions from July 2022 through Sept 2023 (missing 5 sessions for personal or professional reasons). Observations were concluded when data saturation was reached, and no new insights were gleaned from the final 3 observation sessions [37]. AKS recorded jottings and later summarized them in field notes during observations [38]. A review of the field notes was conducted to confirm that no additional novel insights emerged in the final observations.

Observations focused on the technology used in the courtrooms that enabled or created barriers to access. Additionally, special attention was paid to both consumer-facing and provider-facing technologies that impacted each consumer’s mental health care access and retention. This included “social services” and “other services” related to mental health recovery, including housing, substance use treatment, food benefits, vocational support, transportation, and educational services [39].

#### Interviews

AKS conducted semistructured interviews with consumers who were receiving care from CMH and with criminal justice histories (n=14) and health and service providers (HSPs) affiliated with the MHTC (n=14). Prior to conducting the first interview, AKS piloted the interview with a colleague to clarify language and estimate the timing of the interview, with consumer interviews taking 90 minutes and HSP interviews taking 60 minutes. Interviews were conducted using an interview guide. All providers were current or former employees or volunteers who worked with consumers in court. HSP interviews occurred between January 2022 and November 2023, and consumer interviews between October 2023 and May 2024. For all interviews, no one else was present besides the participants and AKS. AKS transcribed the interviews verbatim or summarized the interviews (upon participant request). Interviews for both consumers and providers continued until after the data were saturated and there were no new emergent themes [37]. Subsequently, the final 3 transcripts from both samples (consumers and HSPs) were reviewed to confirm that there were no new emergent findings and that no new inductive themes emerged from the data. No transcripts were returned to participants for comment or correction.

#### Demographic Surveys

Providers and consumers completed a demographic survey after the interviews, with the option to answer survey questions over the phone. Both groups were asked questions about sex, age, race/ethnicity, and the highest level of education. Consumer surveys also asked about patient health insurance and years of technology use.

### Data Analysis

#### Qualitative Data Analysis

All qualitative coding was conducted using NVivo, and iterative review of the transcripts began when data collection started. The coding mixed deductive and inductive approaches. Deductively, AKS and TCV developed codes based on the Levesque et al [16] model, in which each process-oriented concept in the model became a process code. A round of coding was conducted to group various actions and processes across the Levesque framework, specifically coding interviews and transcripts into the following groups: (1) perception or identification of health care needs, (2) health care seeking, (3) health care reaching, (4) health care usage, and (5) health care consequences. While coding the access stages, AKS identified that “staying in care” was an additional stage not accounted for and subsequently added that code, reviewing all transcripts for this additional inductive code. An associated coding matrix can be found in Multimedia Appendix 1 [16]. Furthermore, inductive coding involved AKS and TCV, both researchers with mixed methods health informatics research experience, independently reading 3 randomly selected consumer interviews, 3 randomly selected provider interviews, and 5 randomly selected field notes to develop emergent codes via process coding [40]. To gain a nuanced understanding, AKS triangulated data across the sets of interviews and ethnographic field notes, applying the same codes to all data sources to enable comparison across sources. AKS subsequently developed analytical memos for individual codes to synthesize emergent results and map the inductive codes to the access model.

#### Quantitative Data Analysis

AKS calculated descriptive statistics for demographic variables, including frequencies, means, and SDs.

## Results

### Participant Characteristics

Most consumers (C) interviewed were White and female (12/14, 85.7%), with a mean age of 51.2 years (Table 1). A total of 12 (85.7%) consumer participants had Medicaid or Medicare for health insurance, and 10 (71.4%) were college-educated. In regard to employment, 6 (42.9%) were working part-time, 3 (21.4%) were unemployed, 3 (21.4%) were beneficiaries of Social Security Disability Insurance, and 2 (14.3%) were employed full-time. These consumers were employed full-time and had private insurance due to their positions as peer support specialists at CMH while also receiving CMH care. One consumer currently participated in an MHTC, and 12 consumer interviewees were mental health clubhouse members. Of the clubhouse members, 2 were both consumers and peer support specialists. The HSP participants were primarily White (12/14, 85.7%) and female (10/14, 71.4%), with a mean age of 57.8 years. Service provider respondents held positions at the court, CMH, and housing agencies.

**Table 1.** Demographics of the interview participants (N=28).

Demographics	Consumers (n=14)	Health care and social service providers (n=14)
Sex, n (%)		
Female	12 (85.7)	10 (71.4)
Male	2 (14.3)	4 (28.6)
Age (years), mean (SD)^a^^b^	51.2 (12.2)	57.8 (18.7)
Race/Ethnicity, n (%)^a^		
White	12 (85.7)	12 (85.7)
African American	2 (14.3)	1 (7.1)
American Indian or Alaska Native	1 (7.1)	0 (0)
Hispanic	1 (7.1)	0 (0)
Asian	0 (0)	1 (7.1)
Highest level of education, n (%)^a^		
High school	3 (21.4)	0 (0)
Some college	1 (7.1)	0 (0)
Bachelor degree	3 (21.4)	3 (21.4)
Associate degree	6 (42.9)	0 (0)
Graduate degree	1 (7.1)	6 (42.9)
Professional degree	0 (0)	4 (28.6)
Employment		
Working full time	2 (14.3)^c^	—^d^
Working part time	6 (42.9)	—
Unemployed	3 (21.4)	—
Social Security Disability Insurance	3 (21.4)	—
Insurance^e^, n (%)		
Commercial	2 (14.3)^c^	—
Medicare (Dual Eligible for Medicaid)	8 (57.1)	—
Medicare exclusively	1 (7.1)	—
Medicaid exclusively	2 (14.3)	—
Other^f^	1 (7.1)	—
Years of internet use^e^, mean (SD)	18.1 (8.3)	—

a One service provider did not answer these questions.

b Two consumers did not answer this question.

c These were employed as peer support specialists at community mental health care (CMH) while also receiving CMH care.

d Not applicable.

e Only consumers were asked these questions.

f One consumer had “health care that you bought on health care insurance marketplace” but did not provide further details.

### RQ1. What Changes, If Any, Are Necessary to Improve the Applicability of the Conceptual Framework of Access to Health Care Related to SMI For Low-SES Consumers and Technology-Mediated Care Contexts?

**Figure 1.**
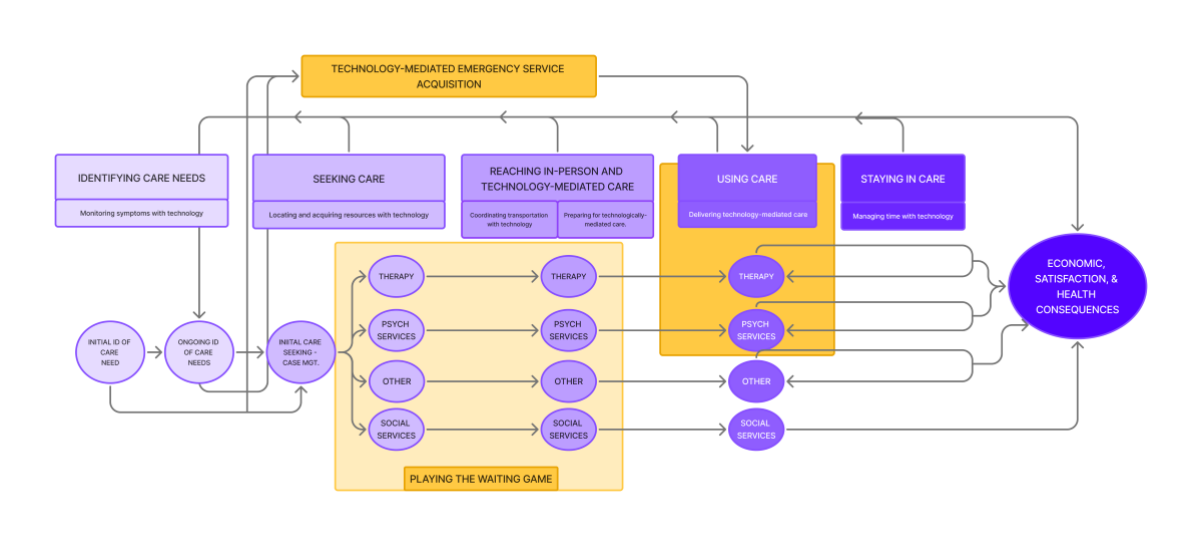
Technologically mediated care access cycle for people with serious mental illness.

We have identified changes necessary to the Leveque et al [16] model and now discuss each stage within the revised model to reflect the empirical data (Figure 1).

#### Perceptions of Needs and Desire for Care

While the Levesque et al [16] model described the “Perceptions of needs and desire for care,” all study participants had already been diagnosed with SMI and were largely using some form of mental health care. Thus, a more apt description of this stage for participants is “Identifying care needs.” Additionally, unlike the Levesque et al [16] model, which categorizes identifying care needs as one discrete stage, there were two need identification stages for study participants. The first stage involved the initial identification of care needs, and the second stage involved the ongoing identification of care needs (Figure 1).

Within this sample, the first stage of initial identification of care needs often occurred decades prior, typically in young adulthood. As one consumer (C012) stated, “I’ve been getting help with mental health since I was younger…I didn’t do that on my own.” Others initially identified needs because of an acute mental health incident in adulthood. For example, C010 stated that “up until the time I cracked, I never needed any [mental health care].”

Some consumers pursued ongoing identification of care needs to become aware of any new or increased needs (Figure 1). Hence, monitoring symptoms with technology was a key part of this stage. For instance, 2 consumers used smartphone mobile apps and wearables in ways that identified symptom changes. As discussed below, technology-mediated communication with friends and family also served this purpose.

#### Health Care Seeking

For the “Health care seeking” stage of the Levesque et al [16] model, care seeking is depicted for only one care setting at once. However, low-SES consumers with SMI frequently sought several different sources of care—some of them related to recovery (eg, housing) but not necessarily providing treatment—often simultaneously. Hence, in Figure 1, we have changed this category to “Seeking Care.” Specifically, for the population under study, this involved therapy and psychiatric services, as well as “other services” and “social services.” Case managers also helped people connect with “other services” based on each consumer’s needs, including substance use treatment, food access, educational services, vocational support, group therapies, and assisted living. This could involve legal services such as probation officers or MHTC staff, depending on the situation. For example, one MHTC participant describes:

*I have a probation officer that really is more of a person that ties the threads between my caseworker [and] my progress in the treatment court. I meet with him every two weeks*.

[C001]

The “social services” that consumers with SMI in this sample regularly sought included: (1) housing resources (ie, Section 8 vouchers or low-income housing), (2) health insurance if needed (eg, Medicaid/Medicare), (3) transportation supports (eg, free bus passes, medical transportation services), (4) assistance with job seeking (vocational support), and (5) food benefits. Typically, “other services” and “social services” were offered by CMH partner organizations. After being connected with these services, consumers could navigate them with varied levels of support. C008 uses CMH for therapy, psychiatry, and case management. Beyond that, he describes some of the different services that CMH helped coordinate, like “$300 towards fixing my car,” securing a “housing voucher,” and obtaining a federally subsidized “food card.”

Another departure from the Levesque et al [16] model was an initial seeking care step: case management. Most consumers experienced this step when they enrolled as their local CMH. When this happened, they automatically received a case manager to facilitate their further care-seeking and linkage. As Figure 1 shows, case management initially facilitated access to psychotherapy, psychiatric services, and necessary “other services” and “social services” that support mental health recovery. CMH typically provides psychotherapy and psychiatric services directly. However, consumers sometimes sought therapists outside the CMH-provided options due to provider shortages, related wait times, and schedule limitations at CMH.

An extension of the technology-mediated care context is locating and acquiring resources with technology. As mentioned, some consumers searched for potential therapists via web-based databases or web searches. Additionally, consumers made inquiries or completed web-based forms on their devices. One consumer described completing a web-based form for multiple resources:

*For my DHHS [Department of Health and Human Services], I just filled out paperwork and then sent that in for my Medicaid and food stamps.*

[C009]

An additional necessary modification of the seeking care stage is the addition of emergency service acquisition, as demonstrated in the revised model (Figure 1); emergency care-seeking circumvented several care access stages when required. This occurred when consumers arrived at psychiatric emergency services of their own volition (calling 911, going to the emergency room [ER]) or due to others in the community calling 911 if they believed that the consumer was a threat to themselves or others. Calling 911 due to a psychiatric emergency may have occurred at either the initial or ongoing needs identification stage. In recognition of the use of technology to acquire emergency services, the category of technology-mediated contact was added. Notably, through this emergency service acquisition, it is also possible that consumers will have contact with the criminal justice system. For example, C003 describes witnessing this experience by saying,

*I have a friend… she gets really sick when she doesn’t take her medicine and the police have to force her to go to the hospital and they have to put her in handcuffs and take her away*.

Calling the police works to keep CMH consumers safe while in crisis, but it often compensates for a lack of resources for low-SES CMH consumers.

*Where there’s a lack [of other crisis care] access, you call law enforcement.*

[HSP07]

Depending on the situation, this step may also result in legal trouble that could lead to participation in the MHTC diversion court.

Starting at the care-seeking stage and continuing through reaching care, a new cross-stage category is needed: “playing the waiting game” (Figure 1). This was a common experience of low-SES consumers with SMI, who often faced prolonged waits for appointments, new provider assignments, housing acquisition, and medical transport. Waiting typically occurred with little guidance or communication of updates from HSPs. For example, a fieldnote from observations at court in April 2023 described the following scenario:

*The judge asked about housing and how this person is doing with their housing situation. They said that it’s not great, but they’re getting by. Money is really difficult and they’re on a waitlist for Section 8 housing.*

Notably, there was typically a lack of technological systems through which consumers could follow the status of applications for housing or other services. Instead, a consumer or case manager sought any updates via email or phone.

#### Health Care Reaching

For the health care reaching stage of Levesque et al [16], we again see low-SES consumers or the HSPs who work with them contacting multiple forms of care, often simultaneously. Furthermore, it is critical to note that the care that was reached was delivered either in-person or in a technology-mediated manner (ie, logging into a computer for a telehealth appointment). Hence, the name given to this stage of the model was altered to say, “Reaching in-person and technology-mediated care.”

Although some care was delivered in person, coordinating transportation with technology for in-person visits was often necessary. Some consumers used transportation apps on a smartphone or booked nonemergency medical transportation. For instance, one consumer (C006) used a bus app to arrive at their appointments on time, stating, “I have maps on my phone, so I use that a lot to tell me when the bus is coming.”

Preparing for technology-mediated care was necessary here as well. This involved consumers acquiring and maintaining technologies, often with the help of HSPs or clubhouse peers. It also consisted of following various procedures and using different technologies driven by those used by the HSP in question. Consumers constantly renegotiate reaching care, partly due to technology-related challenges related to having technology and adapting to various HSP systems. For example, even if a consumer knew how to use a platform (like Zoom), variable settings across providers created complexity.

*With Zoom… sometimes there’s a password and sometimes there’s not a password… so that can get confusing*…*if you’re not consistent with how you’re using something and the rules are changing as you’re getting to different parts of an app or whatnot, I just don’t think it’s fair to people*.

[C004]

C005 also argued that HSPs make “too many assumptions that everyone should be using computers or have the knowledge or capabilities to access everything… one assumption that is that everyone must have patience and endurance to contend with computer glitches, free ups, correction to typos, tech compatibility, and trying to figure out how to use the different apps and how to use the different things accordingly.”

Playing the waiting game also persisted into the health care reaching stage because consumers who lacked personal transportation had to plan their medical transportation far in advance. They also spent much time waiting for transportation providers to arrive. Another participant described how she used her phone to establish medical transportation:

*I usually do it on the app [Access2Care] on my phone. Normally, you could set up transportation within a month in advance.*

[C012]

On the day of the appointment, the transportation service provider often sends a digital notification or reminder:

*When the day comes, I just lie in bed, wait for the ding to give you the text of the drivers on the way. Then, I head out when the driver arrives.*

[C010]

#### Health Care Usage

The Levesque et al [16] model maintains a singular box to represent “health care usage,” with primary and secondary access subcategories. However, this distinction does not entirely fit SMI care for low-SES patients. Rather, here again, there is a need to maintain recognition of the use of multiple services simultaneously: therapy, psychiatric services, other services, and social services (Figure 1). These services were often provided via technology-mediated delivery, such as therapy appointments or court review sessions via telehealth. One benefit of this approach was that it reduced consumers’ travel times by using telehealth calls, making it possible to fit more into their schedules, allowing for increased schedule flexibility. For example, service providers (eg, HSP03) identified such a benefit for MHTC participants (“other services” box in Figure 1), stating that Zoom is “easier for the participants as far as transportation and the ability to get to the court.”

At this stage, there is also a need to recognize the continued pathway of emergency service acquisition. Such direct service occurs via the ER or inpatient care if the hospital admits a consumer. This typically resulted in hospitals discharging patients into CMH care. However, there were situations in which consumers could be court-ordered to adhere to treatment recommendations, often when they posed a risk to themselves or others or if their judgment was deemed to be impaired by their SMI. C003 described her experience with this:

*One time I was in the hospital and they put me on a telehealth thing with a tablet to talk to a court… I was so sick and mentally ill in that hospital that I didn’t know anything that was going on around me.*

[C003]

This pathway primarily stabilized consumers so they could again engage in care through usual routes or make care engagement decisions with sound judgment.

#### Staying in Care (New Stage)

A “staying in care” stage was added to the original framework to accommodate SMI care’s complex access cycle. The importance of this category of access was evident in the experiences of the study participants. Most study consumers received psychiatric services 2‐24 times a year and received therapy as often as every week. The presence of staying in care is an explicit step in the framework to emphasize that consumers benefit from sustained service use and that retention helps produce favorable mental health outcomes from participants’ perspectives. In Figure 1, arrows to the right of the use category indicate that consumers must keep using these services regularly, thus staying in care.

At this stage, managing time with technology is a key adaptation for a technology-mediated care context. This involved accomplishing time-sensitive activities such as scheduling, rescheduling, and remembering health care appointments using various technologies. For example, as discussed subsequently, many consumers used calendaring apps to keep track of their upcoming health care appointments and stay in care. Health care organizations also helped consumers stay in care through reminders in the form of automated phone calls, live calls, text messages, emails, or appointment cards.

#### Health Care Consequences

As for the health care consequences stage*,* the Levesque et al [16] model outlines the economic, satisfaction, and health-related consequences of health care access. While each of these was evident in study results, data concerning the experiences of low-SES consumers suggests the need to modify the framework to additionally reflect the psychosocial and emotional consequences as part of health consequences. Indeed, there were negative consequences in each category above for those who could not figure out how to access technology-mediated care. As we elaborate below, consequences included experiences of frustration, anger, and lowered self-efficacy, suggesting extensions to the existing model. For instance, C004 described how she felt when she could not get her telehealth video call working; she said, “Frustrated. We expect technology to work a hundred percent of the time.”

### RQ2. How Do Low-SES Consumers Use Technologies Throughout the Process of Accessing Health Care Related to Their SMI?

#### Overview

Consumer technology use falls into 3 emergent categories that recur across multiple stages of the access framework: keeping track, figuring it out, and leaning on others. In this study, keeping track entailed consumers monitoring their symptoms, mental health status, medications, schedules, and appointments. This allowed them to make necessary adjustments and clearly understand their present and future care needs. Figuring it out refers to the continuous, individualized efforts of low-SES consumers with SMI to comprehend and navigate their care across multiple services and amid complex and often unclear processes. This involved performing tasks to prevent care disruptions and adapting to new tools used for accessing care. As C006 noted, “Technology in some ways has been good, but it has also been very trying to figure it out.” Leaning on others refers to the external support consumers seek or receive from friends, family, and peers with SMI or HSPs to facilitate access to technologically mediated health care. Consumers leaned on others routinely, such as regularly discussing new symptoms, or on an ad hoc basis, like when troubleshooting spotty Wi-Fi. Having introduced these categories, their presence in the context of each Levesque et al [16] model stage is now outlined.

#### Identifying Care Needs

As mentioned, most low-SES consumers with SMI were primarily engaged in the ongoing identification of care needs rather than in recognizing initial symptoms. At this stage, monitoring symptoms with technology was performed as part of consumers’ larger practices of keeping track. Here, consumers were often leaning on others*,* particularly loved ones or peers, by digitally communicating their SMI symptoms, using tools to identify new or changing symptoms collaboratively. Consumers usually call or text friends and family to “check in” on their mental health. For some, technology-mediated “check-ins” with a loved one increased symptom awareness and allowed consumers to verify their own mental health self-assessments and determine the necessary next steps.

*If I’m in crisis or something I’ll usually reach out to my best friend or my mom or my dad and just check my own thinking on stuff and see how bad it is before I make decision to go to hospital or reach out to [my] case manager.*

[C004]

Regular technology-mediated communication with friends also supported symptom monitoring, allowing individuals to lean on each other for awareness of mental health episodes.

*I have a best friend that I talk to daily…We both notice in each other if we’re having an episode, a bipolar episode.*

[C014]

These mechanisms for technology-mediated communication supported consumers’ needs identification while providing them with social support.

Two consumers also used smartphone mobile apps and wearables in ways that identify symptom changes. Specifically, C009 used mood-tracking features on the MyStrength mobile app designed to self-monitor her symptoms. C008 used a Fitbit wearable and its mobile app for biometric feedback, alerting her to changes in her stress and anxiety: “This Fitbit [that’s] hooked up to my phone… it’ll beep and say, ‘okay, your stress level is at blah, blah, blah’,…it’ll buzz me.” For C008, this app helped her identify her needs, adding that the app helped to make her “more aware.”

#### Seeking Care

Consumers were sometimes proactive in seeking care by figuring out what services were available and how to access them with technological tools. We called this access process “locating and acquiring resources with technology” in the modified framework above. Figuring it out for some consumers involved searching for potential therapists via web-based databases, web search engines, or perusing service-related websites to locate resources. Web-based applications were typically necessary to acquire resources. Some consumers also independently contributed to figuring out how to apply for social services while using technology, such as housing or food stamps, and then did so. Nevertheless, many consumers faced obstacles when figuring out how to access services alone. For instance, issues with insurance compatibility and navigating these websites frequently complicate these processes. Such challenges led consumers towards leaning on others while seeking services, especially HSPs, who guided consumers through the technology-mediated process of applying for services. Importantly, case managers provided a second set of eyes on important paperwork before a consumer submitted it, as one consumer stated*:*

*I filled out the paperwork and let [the case manager] look at it… Just to check for accuracy.*

[C012]

Leaning on others when using technology to seek health care also included others submitting documents related to consumers’ mental health needs. For example, C013 used resources at a mental health clubhouse to fill out and submit a housing application, stating, “The clubhouse staff, they would help me fax applications to different apartments or email them to print them off.” Similarly, C009 stated, “I filled out my paperwork and I think [CLUBHOUSE STAFF] emailed it to the landlord.” By leaning on case managers and clubhouse staff to review and send documents via technology, consumers could more confidently navigate the complex process of seeking social services in technology-mediated ways.

Sometimes, HSPs also offered in-person social support to those who struggled to navigate technology-mediated applications for social services. In turn, this assisted with understanding and emotional regulation.

*I said, 'Oh, you have to fill out this form on the computer.' No, I’m not going to do that. I want to talk face-to-face with somebody because I don’t understand all those forms.*

[C005]

C005 described how in-person case manager support prevented her from becoming too emotionally overwhelmed at the computer: “In-person, if they say, 'Okay, can I save this? Can we break it down to doing it probably 20 minutes at a time.’” By transitioning from an independent digital submission to a supportive in-person approach, consumers found it easier to manage the lengthy and complex form submission processes in the care-seeking stage.

#### Reaching in-Person and Technology-Mediated Care

As mentioned, technology was involved in 2 parts of the reaching care stage: coordinating transportation with technology for in-person visits and preparing for technologically mediated care. For the former, consumers set up their medical transportation through mobile apps or phone calls before their appointment. For example, C006 described reaching in-person services, stating, “I’ve got my [TRANSIT] app and… you schedule rides there.” For some, this coordination included medical transportation services (such as local ride-sharing services for people with disabilities) and ride-hailing services (such as Uber or Lyft).

Figuring out how to coordinate transportation meant that participants had to use various apps or platforms to get to in-person care. Consumers who lived near accessible public transportation often used apps on their cell phones to plan their travel and physically navigate their way to those appointments. For example, one consumer used a bus app to arrive at her appointments on time and also described how she used a website provided by her local transit:

*So I was able to [use] [local bus] in [CITY1], [CITY2], I’m coming from [CITY2], and I take the bus.*

[C003]

As for preparing for technologically mediated care, consumers spent time figuring it out in relation to having the necessary technology for visits. Indeed, consumers often faced financial challenges that restricted their ability to use telehealth as they balanced their health care needs with other expenses. To address this challenge, leaning on others was common. HSPs like case managers and clubhouse staff played a crucial role, providing the technology consumers needed to connect to technology-mediated care. They ensured that consumers had access to the right digital tools and often had private spaces appropriate for conducting telehealth calls.

*I was fortunate. I had Clubhouse, and they were willing to let me be in a room by myself [for the visit] and help me with the Zoom [beforehand]*.

[C009]

Preparing for technology-mediated care also meant acquiring technology skills, another place in which consumers reported leaning on others. For instance, one consumer who had a peer support role at a Clubhouse also described her role in teaching her peers how to use novel technologies:

*I did take a couple of tablets to specific members that did not have any technology experience. So I had to show them how to start it up, how to get to the program, how to connect with virtually… it was a training process.*

[C008]

Furthermore, as mentioned, it was common for each consumer to have multiple providers with whom they interacted regularly. Often, the consumer had to figure it out in relation to interacting differently with each provider, either via different technologies or in person. Due to variable mechanisms for engaging with other HSPs, it was difficult for consumers to know what was appropriate and what was not, necessitating figuring it out. As one consumer described, mechanisms for accessing health information were not streamlined. This puts consumers in a position to try multiple digital methods (eg, calling and portal use) to change or reschedule an appointment. C006 explained how she would get a new appointment scheduled, expressing frustration with the process:

*Probably I would try to call first, and then if I can’t get through, then I would probably go on the portal and try to schedule an appointment. But again, if it’s something new, [technology] takes me a while to learn.*

Leaning on others also taught consumers how to use technology-mediated communication to interact with service providers. A peer support specialist and consumer (C004) stated, “I can show people how to email their case manager or get on the phone and teach them proper etiquette.” This effort needed to figure it out, which added complexity to reaching care.

#### Using Health Care

Health care typically involves some technology-mediated care delivery. For example, C012 described the range of services he used:

*I go to therapy at least twice a month, and I have a case manager that I see once a month and a psychiatrist. I see him every three months, two to three months. And most of our appointments are in-person, but some of my therapy sessions are over Zoom or telehealth*.

Notably, for this consumer and others, the provider typically decided whether an appointment would be in-person or technologically mediated, often without much input from the consumer. When it came time for telehealth visits, consumers reported leaning on others, specifically clubhouse staff and members, to make visits work. In clubhouses, there was an environment in which learning to use or access technologies for care was a collaborative effort. One consumer shared her experience, stating:

*It was a bit confusing to me because my phone does beat me up sometimes, and so I had to have [Clubhouse Staff] come in and help me go to my text messages, I think, and click on the [TELEHEALTH] link.*

[C013]

At times, clubhouse members also walked other members through using a novel device to connect with care.

Keeping track of medications and appointments was a goal of some SMI consumers, and it was especially important for consumers involved in the MHTC since therapy was part of probation conditions. However, this was difficult for most consumers. Regardless of MHTC involvement, there was a lack of sufficient consumer-facing technologies. Indeed, most consumers in this study did not have patient portals for their CMH care. This stymied consumers. As C004 mentioned, “I use the patient portal for [HEALTHCARE SYSTEM], so I should probably use it for CMH too.” Limited or no access to their records made it burdensome for consumers to obtain information about diagnoses, medications, and appointments. Hence, consumers were trying to figure out how to manage their medications without efficient technological systems for medication management and how to keep track of appointments without such technologies. Consumers saw a need for improved technological systems that would enable them to track and manage their care easily. C002 described the potential benefits of having an integrated portal:

*Because then they can see [their medical information] in front of their face… instead of having to rely on memory or texts or phone calls and messages. It’s right there in front of you… it’s empowering just to have that access.*

As a result of inadequate access to personal health information, consumers often had to lean on others. Specifically, they relied on case managers as intermediaries to keep track of their appointments and medications. C002 explained how she would obtain her medication list:

*I would start with my case manager. She probably would refer me to somebody in records... I might try saying, 'Hey [CASE MANAGER], can you have [PSYCHIATRIST] send a med list so you can send it to me?' I just want to see what’s on my list*.

#### Staying in Care

A major part of staying in care is managing time effectively to remember and attend appointments. Service providers highlighted that some consumers face significant adjustments when beginning or reinitiating care. An MHTC HSP (HSP08) noted that consumers struggled to establish “an understanding of all that is expected to them and time management and being able to comply with [APPOINTMENTS].” Additionally, HSP04 stated:

*Some of them…clearly haven’t even had experience keeping a calendar before… that is often a big adjustment time, that it’s hard for them to pull it together.*

Thus, some consumers had to figure out how to manage time with technology to stay in care.

From those figuring it out to those who were more accustomed, consumers in the sample used calendaring apps to keep track of their upcoming health care appointments to stay in care. One consumer (C004) said, “I use the calendar app a lot because if I don’t put it in there and get a reminder, I’ll forget about.” Knowing the different upcoming commitments ensured that they could attend and prepare themselves for these appointments. To keep track of her medications, one consumer (C008) also used a “reminder list on my phone that says, ‘okay, take this medication with you today.’”

While digital calendars worked for some, other consumers used physical calendars to keep track of appointments and other commitments. Still, others used both digital and physical calendars to reinforce their memory by recording upcoming appointments in multiple places. When asked how she used her paper calendar, one consumer stated

*[For] people’s birthdays and my med reviews and doctor appointments with my primary care physician and stuff like that.*

[C003]

Consumers with challenges attending appointments also used physical calendars to keep track, as indicated by this MHTC fieldnote:

*The judge mentions that [CONSUMER] has missed a few meetings with CMH and asks him what is going on with that. He says not that he knows of. He doesn’t think he has missed any therapy appointments… He mentioned that he bought a physical calendar because he’s been having trouble keeping track of things. He mentioned he doesn’t want to have to rely on his phone but it’s a work in progress.*

This consumer was reticent to adopt a digital calendar but ended up incorporating both versions for keeping track.

Consumers reported leaning on others through digital and paper reminders provided by health care organizations and the court to help them stay in care. For instance, health care organizations help consumers stay in care through reminders in the form of automated phone calls, live calls, text messages, or emails. This resulted in consumers keeping track of appointments more easily. One consumer described her experience with phone call reminders:

*They [CMH] still do old school automated phone reminders. So you answer and they say, ‘Hello, you have an appointment with [NAME].’ I go on whatever date at two o’clock.*

[C002]

This kind of reminder from CMH helped jog consumers’ memory, alerting them that they needed to attend an appointment—often the next day. Another consumer noted they are less likely to miss care when they receive reminders:

*[CMH] sends me reminders about my appointment… that helps a lot because sometimes I wrote it down wrong on the calendar at home or I haven’t wrote it down or something like that.*

[C013]

In other settings, including the MHTC, consumers were also given a paper card or sheet indicating their upcoming appointments to help them keep track of appointments. This is analogous to paper reminder cards that consumers receive in person after health care appointments:

*I do get a reminder card from them when I go, and most of the time I keep it in my wallet… if I’m not sure what time it is, I’ll go back and look at the card though.*

[C012]

One service provider also described how case managers often gave such physical reminders:

*We’re like, ‘Hey, this is just as a reminder. So, you know, it’s coming up, they might wanna put it out in their fridge or in their wallet or purse*.’

[HSP02]

Consumers who could not figure it out with respect to managing time with technology risked missing care-related appointments, even if an MHTC mandated them. An example of this in the legal context can be seen in MHTC fieldnotes:

*I observed an MHTC consumer struggling to try to connect to the court via Zoom at that moment and described how they missed an appointment because of phone issues*, *‘ I know you’re connected; I can see your screen, but something’s wrong… ’ said the judge, trying to figure out what was happening. The consumer lets the judge know that his phone went out; he can’t see its screen, so it’s hard to maneuver. He almost missed court because he couldn’t get it to work; he missed another one of his appointments because his phone wasn’t working right*.

#### Care Consequences

The original Levesque model highlights the economic consequences of care, which, for our purposes, encompasses the time and travel costs related to health care visits. For technology-mediated care, consumers highlighted positive impacts on time costs. C004 explained:

*Even people that can’t make it in for meetings, they’re able to hop on Zoom and join meetings, which is so important for so many people*.

Reduced transportation challenges were also evident from consumers’ perspectives. C014 said that technology-mediated connections remove additional time needed to get to care: “I don’t even have to think about traveling or taking the bus, it’s easier to do it virtually.” HSPs identified a similar benefit for MHTC participants, stating that Zoom is:

*Easier for the participants as far as transportation and the ability to get to the court.*

[HSP03]

Satisfaction is also a health care consequence in the Levesque et al [16] model. Most consumers advocated for maintaining hybrid care options to increase access, recognizing both the benefits and trade-offs of technologically mediated care and improving overall satisfaction. Here, it was recognized that technology-mediated care could make people more comfortable as an aspect of satisfaction. For example, HSP05 mentioned that when conducting court sessions over Zoom, consumers are “more comfortable. It’s not as intimidating.” HSP02 also reflected this sentiment:

*Telehealth allows [consumers] to be in the comfort of their own home and not have to navigate bus routes, making it much more comfortable for them*.

However, less-satisfied consumers and service providers raised concerns about negative impacts on social connectedness, with C003 stating, “It just kind of makes me feel lonely seeing people in a computer.”

The Levesque et al [16] model also highlights the health consequences of care, including emotional and psychosocial health, for this study. Here, the impacts of technology-mediated care could be negative. Specifically, failing to figure out how to facilitate technology-mediated care delivery caused emotional distress, as one peer described:

*Not knowing how to do something or not having the monies to afford a more expensive computer... adds to the whole self-blame*.

[C005]

This may lead to frustration and anger, as a consumer noted:

*If I don’t succeed, I get very angry with myself.*

[C006]

## Discussion

### Principal Findings

For low-SES consumers with SMI in the United States, the process of accessing care departs from the Levesque et al [16] model in several key ways. The revised model reworks the identification of care needs to include initial and ongoing stages. There were also 3 approaches to accessing care not outlined in the Levesque et al [16] model: emergency services, initial care access enabled through case management, and subsequent and often simultaneous care through various services (therapy, psychiatric services, other services, social services). For this study sample, reaching care also involved in-person and technology-mediated services. Moreover, the addition of staying in care was validated by empirical results. Care consequences also involved recognition of emotional and psychosocial health. The revised model incorporates roles for technology throughout the access stages indicated in the purple rectangle in Figure 1): monitoring symptoms with technology (identification of care needs)*;* locating and acquiring resources with technology (seeking care)*,* coordinating transportation with technology and preparing for technologically mediated care (reaching in-person and technology-mediated care)*,* technology-mediated care delivery (using health care)*,* and managing time with technology (staying in care). Technology-mediated contact was also present for emergency service acquisition.

Consumers’ use of technologies thus influenced every stage of their access to health care. Key use categories included figuring it out, leaning on others, and keeping track. For leaning on others in technology use, consumers reported relying on friends and family to help monitor and track symptoms, apply for services, make telehealth visits work, and learn to connect digitally. This aligns with work that highlights how justice-involved individuals, such as those who were formerly incarcerated, often leverage offline networks to navigate an increasingly digital world [41]. Figuring it out involved learning to acquire services independently, coordinating transportation with technology, receiving care from HSPs across multiple platforms, and developing the ability to engage in managing time with technology. Together, these practices helped to ensure that consumers attended scheduled appointments, thus allowing them to stay in care over time.

### Comparison With Prior Work

Previous studies have described numerous technologies designed to identify mental health care needs. For instance, prior literature outlines diagnostic tools, self-monitoring technologies for tracking symptoms, sensing technologies, and algorithmic detection [42-47]. However, while 2 participants engaged with apps for monitoring symptoms, more people in this study monitored symptoms via technology-mediated discussions with friends and family. Specifically, consumers called or texted their supportive loved ones and used these interpersonal relationships as barometers for evaluating their mental health status, either explicitly (asking a friend for advice) or through mutual passive monitoring. Therefore, the findings of this study uncovered new relational methods for self-monitoring symptoms with technology.

The need for multiple services and the lack of the service organization’s clarity about how to pursue them often overwhelmed consumers. Participants frequently did not have adequate technology to submit service applications independently, nor did they know how to fill them out. Consequently, study consumers often needed the support of their case managers or peers to complete web-based applications for housing or disability benefits, such as those consumers receiving Social Security Disability Insurance. While prior work has identified the importance of case management in helping clients fill out applications for benefits [48], this work is novel in highlighting how this type of support may be particularly necessary with a shift to technology-mediated platforms. When technological literacy was a prerequisite for applying for public benefits and assistance, HSPs’ roles evolved to encompass cofacilitating those applications.

Adding the emergency services acquisition model based on study results highlights direct, technologically mediated pathways to care acquisition. This aligns with other studies that identified that populations with low SES have increased rates of 911 calls and psychiatric ER use due to mental health emergencies. The observation of increased rates of ER use among low-SES populations compared with higher-SES populations is also found in other health conditions [49]. Emergency services sidestepped the other cycle stages while sometimes problematically engaging with law enforcement during entry to care. These data align with the findings of Wood et al [50] of the “gray zone” of policing, asserting that those with unmet mental health needs are “unengaged or under-engaged individuals who fall through service cracks.” This aligns with findings around there being multiple points at the access continuum wherein consumers can drop out of care, thus not staying in care or being retained. While this safety net is helpful in emergencies, more work needs to be done to prevent the escalation of mental health concerns from reaching emergent levels. Investments in programs that provide ambulatory psychiatric assessments and care, such as mental health urgent care, may help to decrease the need for emergency services. One example that successfully leveraged technologies was the Veterans Health Administration’s efficacious implementation of unscheduled emergency telehealth interventions within urgent care clinics [51]. This is increasingly important when considering the intersection of race, SES, and mental health status, as Black individuals with SMI are less likely to receive any mental health care [14] and face increased risks of lethal altercations with interactions with law enforcement [52].

This work also contributes novel findings that show that services such as mental health clubhouses can facilitate access to technology-mediated care. Specifically, members often act as “informal peers,” stepping in to provide support as needed. The value of this type of technology-focused peer support has also been observed in other fields, such as oncology [53]. Relatedly, there has been an increasing investigation into “digital peer support,” or the delivery of peer support via technology [54]; however, this work affirms that some consumers require in-person support [55]. Future work should investigate how best to arrange peer technology support services for diverse consumers and situations.

### Theoretical Implications

One key contribution of this work is its extension of the health care access framework of Levesque et al [16] to better represent technologically mediated mental health care access for low-SES CMH consumers (Figure 1). By establishing the cyclical nature of mental health care access for low-SES consumers with SMI, this extends the original framework of Leveque et al [16] to encompass situations more likely to emerge with other chronic and complex conditions. For example, cyclical patterns in accessing care likely extend to other chronic conditions such as HIV, postural orthostatic tachycardia syndrome, or asthma, which share the need for ongoing and often lifelong management to maintain wellness [56-58].

The extended model also shows that health care often involves multiple forms of care at once. Hence, the care access stage for one service (eg, seeking stable housing outside a shelter) may be at an entirely different stage than that for another service (eg, obtaining biweekly therapy sessions from a CMH therapist). A consumer may thus experience multiple access stages simultaneously, as the stages are not mutually exclusive to one type of service. Moreover, consumers with SMI typically need long-term care to manage symptoms, and they may move in and out of care with different providers or perhaps cease some types of care altogether for a period of time or permanently.

This extended model also considers consumer retention in care as a vital yet often overlooked aspect of health care access. Staying in care can result in positive patient outcomes, leading to recovery or better symptom management. On the other hand, not staying in care or experiencing care attrition can lead to deteriorating health outcomes [59,60]. While staying in care is crucial for managing SMI, health care needs can change over time. For example, a person with a stable SMI and existing social services linkages might have monthly therapy appointments and biannual psychiatric appointments for medication management. In contrast, individuals who experience acute SMI symptoms may require more active symptom monitoring and additional services.

Another novel aspect of the extended model is the addition of roles for technology throughout each step in the process of access to care. Specifically, the purple box below the access cycle displays the role of technology throughout the different steps, including technology monitoring symptoms, locating and acquiring resources, preparing for telehealth and coordinating transport, delivering technology-mediated care, and supporting time management. This technology-enhanced process likely generalizes to chronic health conditions, as telehealth is increasingly available after COVID-19 care. Researchers must continue investigating hybrid and digital care models for low-SES populations beyond those with SMI [61].

The model visually displays the complexity of accessing care for SMI, laying out ongoing processes that must be managed simultaneously. Notably, technology-mediated care has largely added to this complexity for consumers. Together, these factors can challenge consumers, primarily when they are symptomatic. As such, this model illuminates the complicated patient journey through care and can equip consumers and HSPs with a blueprint for assessing where individual consumers may need additional support in the care access cycle. Ultimately, this revised framework can be used to map journeys for care access and highlight areas needing support. Specifically, service providers could use this tool to assess difficulties in journeys to health care access for individual patients. For example, for consumers who struggle with transportation, providers may be able to identify additional means to support them in reaching care, potentially preventing barriers before they arise.

Furthermore, the expanded framework could be used at the system level, unearthing issues such as prolonged wait times (“playing the waiting game”) and stages at which consumers are likely to fall out of care in local care systems. For example, suppose consumers are largely able to stay in psychiatric care, but there are high rates of dropping out of therapy. In that case, interventions can be developed to target improvements within that specific stage (staying in care) for that particular service (therapy). By breaking down the concept of access into more granular stages, clinical care can more precisely allocate resources to access stages needing additional support.

### Clinical Implications

This study affirms prior research indicating that the abrupt shift from primarily in-person services to telehealth services in the wake of the COVID-19 pandemic may have disproportionately negatively impacted care access for low-SES consumers [27,62-64], including preliminary related work [65]. In this study, those without technological resources and skills faced additional barriers to using technologies across access stages, which aligns with recent SAMHSA telehealth guidelines that detail usability and functionality barriers that impede care access [66]. Providers thus have an opportunity to better understand the digital connectedness of their consumers and work to accommodate them. To do so, health systems could conduct a digital readiness assessment upon intake to assess consumers’ abilities to engage in technology-mediated care. To do so, providers may want to adopt tools such as the “Telehealth readiness assessment tool from the Maryland Health Care Commission” [67] or the “Digital Health Readiness Questionnaire” [68]. This would improve both awareness of individual consumer needs and organizational readiness for telehealth implementation, which has been identified as a major challenge by SAMHSA in the treatment of consumers with SMI [66]. In addition, the results of this study support the efforts of Bobert et al [69] to advocate for assessments that match the dynamic nature of technology-mediated care access. It is crucial to understand if consumers can access care digitally and what support they need, or provide alternative strategies for care access if consumers lack the ability or interest in technology-mediated care.

This work unearths the complicated nature of obtaining technology-mediated care, a significant issue. Clinical settings can address this by transparently guiding consumers on navigating care, potentially through support personnel such as digital navigators who provide digital access assistance. Digital navigator programs train staff to promote digital literacy skills in clinical settings, supporting consumers in learning the skills needed to navigate digital care access in culturally appropriate ways [70,71] While peer support specialists in this study started providing digital navigation services in an emergent way to meet the needs of their fellow consumers, expanding peer support specialists’ roles to officially include technology instruction could enable peers to integrate these services into their formal responsibilities. This programming type should follow the principles set out by Wisniewski et al [72], such as core smartphone skills, technology troubleshooting, language specific to digital health, and approaches to support digital engagement. Furthermore, these peer technology specialists could provide both scheduled and on-demand support, helping consumers troubleshoot issues, set up initial accounts, practice using telehealth platforms before appointments, and gradually build consumers’ independence through scaffolded learning experiences [72]. Checklists that outline care expectations provided digitally or in print, per the consumer’s preference, would help create a more centralized approach to building consumers’ understanding of digital care. In addition to social services, this checklist could also be generated for available providers such as therapists and psychiatrists.

Beyond peer support, accessible hybrid care involving both digital and face-to-face elements can potentially be enhanced through policy changes. Local and national policies should focus on developing policies that enable low-SES consumers to continue engaging in hybrid care with less emotional and financial strain. Furthermore, the CARES Act introduced temporary flexibilities for telehealth reimbursement, and while this provision was temporarily extended through September 2025 [73], this study provides evidence for the continuing need for telehealth reimbursement flexibilities, especially within a low-SES consumer population. There have been recent moves by the US Federal government to expand telehealth services for nonbehavioral health care [74], and such policies should begin to integrate behavioral health as well.

### Limitations

There are several study limitations. The interviews and observations were conducted in one midwestern state within the United States, potentially reflecting local norms regarding care access. While this may impact generalizability, there was a diverse age range of consumers, although consumer participants did not reflect the broad racial diversity of CMH participants. While most consumers were on Medicaid and were not employed full-time, they were also highly college-educated, meaning they may have had more advantages than some low-SES consumers. With demographic differences in mind, there was little mention of the intersections between SES, race, and SMI within the interviews, which should be further researched. Additionally, consumers who can be interviewed may be more stable in their recovery, and further research is needed to understand whether consumers in less stable recovery use technologies differently. Service providers’ perspectives on retention in care may be influenced by supporting MHTC consumers who may adhere to care to avoid judicial consequences; nevertheless, the consumer interviews provide insights into care retention for consumers not legally obligated to engage with care, providing a well-rounded consumer perspective. Furthermore, this study does not provide a quantitative validation of the framework extensions, which could be a focus of future work. Regarding qualitative data analysis, although there was dual code generation and regular meetings to discuss the coding approach, a formal assessment of interrater reliability was not conducted. Finally, most of the consumers in this study participated in their local clubhouse (n=12), which may account for the frequency with which peer support emerged as an important factor in CMH care access.

### Conclusions

This study proposed a new model of a technology-mediated mental health care access cycle for low-SES CMH consumers that demonstrated the cyclical and ongoing nature of care access for this population. It also showed that retention, or staying in care, is critical to the access cycle. Technology use shapes access to mental health care for low-SES consumers with SMI, with roles for technology at every stage of care access. Findings highlighted consumers’ ongoing struggles to figure it out despite navigating multiple services, and with a lack of technology-mediated tools like portals. Results also underscore the importance of social connections in enabling access to technology-mediated care, notably as case managers and peers give consumers someone “to lean on” when using technology. Future work should investigate the ways in which technology-mediated care can make care access easier rather than harder. As one study participant said, technology-mediated health care “should not be only available to people who can go through all those steps. It should be available to everybody.”

## Supplementary material

10.2196/79608 Multimedia Appendix 1 Coding matrix.

10.2196/79608 Checklist 1 COREQ checklist.

## Abbreviations

CMH: community mental health care
COREQ: Consolidated Criteria for Reporting Qualitative Research
ER: emergency room
HSP: health and service provider
MHTC: Mental Health Treatment Court
SES: socioeconomic status
SMI: serious mental illness
