# Supplementary material for: Access to Technology-Mediated Community Mental Health Care Among Low-Socioeconomic Status Consumers With Serious Mental Illness: Qualitative Study
Source: JMIR Form Res. 2026 Apr 20;10:e79608. doi: 10.2196/79608 (PMC13095047; doi:10.2196/79608)
Supplement: Multimedia Appendix 1 [file formative-v10-e79608-s001.docx]

Supplemental Appendix 1 - Coding matrix matching original theoretical constructs with adapted categories.

| **Access Stages in paper model** | **Originating Source - Original Category** | **Adaptations from Original Source** | **Examples** |
| --- | --- | --- | --- |
| Identifying Care Needs | Levesque et al. Model^1^ - originally titled “Perceptions of needs and desire for care” | Added two sub stages:  (1) initial identification of care needs and  (2) ongoing identification of care needs | (1) "I've been getting help with mental health since I was younger…I didn't do that on my own." [C012]  (2) *"I have a best friend that I talk to daily…We both notice in each other if we're having an episode, a bipolar episode."* [C014] |
| Seeking Care | Levesque et al. Model^1^ - originally titled “Health care seeking” | Added two substages:  (1) initial care seeking, often through case management and  (2) ongoing care seeking of individual services. For ongoing care seeking, therapy, psychiatric services, social services, and other services emerged as relevant subcategories. | (1) Field notes indicated that participants automatically received a case manager to facilitate their further care-seeking and linkage.  (2) A consumer describes getting support in the process of seeking new services,” *"I filled out my paperwork and I think [CLUBHOUSE STAFF] emailed it to the landlord*." [C009] |
| Reaching In-Person and Technology-Mediated Care | Levesque et al. Model^1^ - originally titled “Health care seeking” | Reaching care was bifurcated into reaching (1) in-person and (2) technology-mediated care. Furthermore, the types of care services that individuals reached were also split into the four aforementioned service categories. | (1)“*I've got my [TRANSIT] app and… you schedule rides there.” [C006]*  (2)”*they were willing to let me be in a room by myself [for the visit] and help me with the Zoom [beforehand]*.” [C009] |
| Using Care | Levesque et al. Model^1^ - originally titled “Health care utilisation” | Using care maintained the four category division seen in other categories. Furthermore, while the original model shows a single “utilization” point, our data indicated that consumers regularly were cycling through the use of care, with different cadences for different services. | C012 described the range of services he used: “*I go to therapy at least twice a month, and I have a case manager that I see once a month and a psychiatrist. I see him every three months, two to three months”* |
| Staying in Care | *Emergent from the data* | *NA* | A provider describes the processes involved in ‘staying in care’ *“Some of them…clearly haven’t even had experience keeping a calendar before… that is often a big adjustment time, that it’s hard for them to pull it together.”* |
| Health Care Consequences | Levesque et al. Model^1^ - originally titled “Health care consequences” | The category of health care consequences was maintained, however, it was adapted to reflect the circular nature of mental healthcare access. The consequences of technology-mediated access often saved time, but at times led to consumer distress. | Positive consequence: “*Telehealth allows [consumers] to be in the comfort of their own home and not have to navigate bus routes, making it much more comfortable for them*.”  Negative Consequences: “*Not knowing how to do something or not having the monies to afford a more expensive computer... adds to the whole self-blame.*” [C005 |
| Model Additions | |  |  |
| Technology Mediated Emergency Service Acquisition | *Emergent from the data* | *NA* | *"Where there's a lack [of other crisis care] access, you call law enforcement."* [HSP07] |
| Playing the waiting game | *Emergent from the data* | *NA* | “*Money is really difficult and they're on a waitlist for Section 8 housing." [Fieldnote]* |

^1^ Levesque J-F, Harris MF, Russell G. Patient-centred access to health care: conceptualising access at the interface of health systems and populations.Int J Equity Health. 2013;12:1-9. doi: 10.1186/1475-9276-12-18
